# Love Wave Propagation in a Piezoelectric Composite Structure with an Inhomogeneous Internal Layer

**DOI:** 10.3390/ma19061151

**Published:** 2026-03-16

**Authors:** Yanqi Zhao, Peng Li, Guochao Fan, Chun Shao

**Affiliations:** 1 School of Mechanical Engineering, Hangzhou Dianzi University, Hangzhou 310018, China; 2 State Key Laboratory of Mechanics and Control for Aerospace Structures, College of Aerospace Engineering, Nanjing University of Aeronautics and Astronautics, Nanjing 210016, China

**Keywords:** Love waves, phase velocity, electromechanical coupling factor, piezoelectric damage

## Abstract

An inhomogeneous thin internal stratum sometimes exists between two dissimilar materials, which is usually caused by non-uniform thermal distribution, interaction of different media, diffusion impurity or material degeneration and damage. In this paper, it is considered as a functional graded (FG) piezoelectric material in surface acoustic wave devices, and we investigate its effect on Love wave propagation within the framework of the linear piezoelectric theory. Correspondingly, the power series technique is presented and applied to solve the dynamic governing equations, i.e., two-dimensional partial differential equations with variable coefficients, with the convergence and correctness being proved. In this method, the material coefficients can change in random functions along the thickness direction, which reveals the generality of this method to some extent. As the numerical case, the elastic coefficient, piezoelectric coefficient, dielectric permittivity, and mass density change in the linear form but with different graded parameters, and the influence of material inhomogeneity on the Love wave propagation is systematically investigated, including the phase velocity, electromechanical coupling factor, and displacement distribution. In addition, the FG piezoelectric material caused by piezoelectric damage and material bonding is discussed. Numerical results demonstrated that both piezoelectric damaged and material bonding can make the higher modes appear earlier for the electrically open case, decrease the initial phase velocity, and limit the existing region of the fundamental Love mode for the electrically shorted case. The qualitative conclusions and quantitative results can provide a theoretical guide for the structural design of surface wave devices and sensors.

## 1. Introduction

The Love wave, established by A. E. H. Love in 1911, plays an important role in surface acoustic wave (SAW) devices, including filters, delay lines, oscillators, amplifiers, and so on [1,2,3,4]. It has only one shear horizontal displacement component, which perpendicular to the propagation and penetration directions and travels in a thin sensitive layer deposited on a substrate when the wave speed is larger than the bulk shear velocity in the layer and smaller than that in the substrate [5,6]. The penetration is comparable with the wavelength, and the energy mainly concentrates on the surface, which leads to its high mass sensitivities at a certain frequency and is especially suitable for liquid sensors [2,6,7].

In order to improve the properties of Love waves, a stratum of finite depth has been added as an internal middle layer in the layer/substrate composite structure mentioned above [8,9,10]. For example, to avoid defects and brittleness, an extra piezoelectric ceramic or polymer layer is embedded between the layer and the substrate [8]. Additionally, an additional thin SiO_2_ [9,11] layer has been inserted as a middle layer to realize a relatively low or even zero temperature coefficient of delay, which can efficiently improve SAW device stability.

From the perspectives of device fabrication, an internal layer sometimes cannot be avoided due to various causes, such as non-uniform distribution of temperature, interaction of different media, diffusion impurity, piezoelectric damage, and so forth [12]. In addition, when bonding the layer on the substrate surface, an interphase or transition, with a thickness typically in the range of 30–240 nm, exists across the interface [13,14,15]. For instance, a homogeneous internal epoxy-bonded layer was predicted in a copper-aluminum layered specimen by Wu et al. [16,17], and its effect on laser-generated surface wave dispersion was investigated. However, the bonding layer in these previous works [13,14,15,16,17,18,19,20] is usually considered homogeneous, with its material properties keeping constant along the thickness and length direction. Due to damage, interface connection or other external factors, once this layer occurs, it is difficult to maintain the homogeneity of the material properties of the internal stratum. Hence, it is essential to investigate the influence of an inhomogeneous internal stratum caused by material damage or interface connection on wave propagation, which is the innovation of this contribution.

For waves in an inhomogeneous or functional graded (FG) piezoelectric material, the mechanical field is coupled with the electrical field, which is hardly decoupled. This is because the dynamic governing equations belong to second-order partial differential equations with variable coefficients. Solving this kind of equation directly is a difficult task. The Wentzel–Kramers–Brillouin method can be used to solve it. But this method is only suitable for large wave numbers [6,21,22]. The stiffness matrix method [23,24], state-space method [25,26], reverberation-ray matrix method [27,28], Legendre polynomial approach [29,30] and hybrid numerical method [31] can be used to solve it. However, these methods are based on the transfer matrix method, in which the inhomogeneous layer is divided into many sub-layers in advance and the material is assumed homogeneous in these sub-layers. The computational efficiency is dependent on the number of sub-layers, and the computing process is very complex. In order to solve the second-order partial differential equations with variable coefficients caused by material inhomogeneity, the power series technique is presented, with its convergence and correctness further validated theoretically and numerically, which is another innovation of this paper. To some extent, this method is general, with the material parameters changing arbitrarily, and the solution is available for any wave number without any limitation.

Firstly, the description of the problem and Love wave solutions are presented in Section 2. After the correction and convergence are proved, the effect of functional graded parameters is systematically discussed in Section 3. Subsequently, an inhomogeneous stratum caused by piezoelectric damage and a transition buffer layer is investigated as the potential engineering application. Finally, some conclusions are drawn. Considering that the material tensors of crystals with 6 mm symmetry possess the same structures as polarized ceramics, the present method and analysis are still valid for 6 mm piezoelectric crystals, e.g., ZnO and AlN.

## 2. Statement of the Problem and Solutions

**Figure 1:**
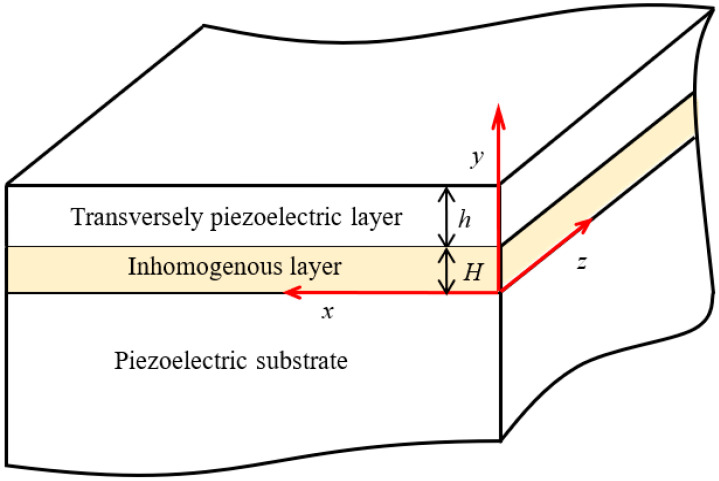
Schematic of the composite structure.

An SAW device usually consists of two layers, i.e., an upper piezoelectric sensitive layer and a substrate. The piezoelectric sensitive layer is so thin that the substrate is viewed as a half-space for analysis. Therefore, we consider wave propagation in a three-layer composite structure consisting of a transversely piezoelectric sensitive layer and a piezoelectric half-space substrate with an inhomogeneous internal stratum layer, as shown schematically in Figure 1. This inhomogeneous internal stratum is considered as an FG piezoelectric material layer. For the piezoelectric medium, the linear piezoelectric constitutive equations can be expressed as follows [32,33,34,35].

(1) $$\left\{\begin{array}{l} \sigma_{ij}=c_{ijkl}u_{k,l}+e_{lij}\varphi_{,l},\\ D_{i}=e_{ikl}u_{k,l}-\varepsilon_{il}\varphi_{,l}, \end{array}\right.$$

where *σ_ij_* and *D_i_* are the stress tensors and electrical displacement vector. *c_ijkl_*, *e_lij_*, *ε_il_* are the elastic coefficients, piezoelectric coefficients, and dielectric permittivity, respectively. ***u*** is the displacement vector, and *φ* represents electrical potential function. An index after a comma denotes partial differentiation with respect to the coordinate. For the piezoelectric materials poled in the *z* direction in Figure 1, *h* and *H* are the thickness values of the top layer and non-uniform middle layer, respectively. The propagation of Love waves may be represented by displacement components, and the electrical potential function as follows [6,8,9,12,19].

(2) u=v=0, w=w(x,y,t), φ=φ(x,y,t).

Thus, the governing equation can be expressed as follows [19,36,37].

(3) $$\begin{array}{l} c_{44}\nabla^{2}w+e_{15}\nabla^{2}\varphi =\rho \ddot{w},\\ e_{15}\nabla^{2}w-\varepsilon_{11}\nabla^{2}\varphi =0. \end{array}$$

where *ρ* is the mass density, ∇2=∂2∂x12+∂2∂x22 is the two-dimensional Laplace operator, *t* is time, and the dot means time differentiation.

### 2.1. Solutions for the Substrate y < 0

Here, we use the star and prime symbols to distinguish the different parameters in the substrate *y* < 0 and sensitive layer *H* < *y* < (*H* + *h*), respectively. Given that the displacement and the stress are finite when *y*⟶∞, the solutions for the Love wave propagation in the positive *x* direction can be expressed as

(4) $$\left\{\begin{array}{l} w^{*}=A_{1}^{*}e^{kb^{*}y}\exp\left[ik(x-ct)\right],\;\\ \varphi^{*}=(\frac{e_{15}^{*}}{\varepsilon_{11}^{*}}A_{1}^{*}e^{kb^{*}y}+B_{1}^{*}e^{ky})\exp\left[ik(x-ct)\right], \end{array}\right.$$

where $A_{1}^{*}$ and $B_{1}^{*}$ are undetermined constants, $b^{*}=\sqrt{1+{\left(c/c_{sub}\right)}^{2}}$, and *k* is the wave number in the *x* direction. $c_{sub}=\sqrt{{\bar{c}}_{44}^{*}/\rho^{*}}$ is the bulk shear velocity in the substrate with ${\bar{c}}_{44}^{*}=c_{44}^{*}+\frac{{e_{15}^{*}}^{2}}{\varepsilon_{11}^{*}}$. Using Equation (1), the stress component $\sigma_{zy}^{*}$ and electrical displace $D_{y}^{*}$ can be obtained as

(5) $$\left\{\begin{array}{l} \sigma_{zy}^{*}=\left(kb^{*}{\bar{c}}_{44}^{*}A_{1}^{*}e^{kb^{*}y}+ke_{15}^{*}B_{1}^{*}e^{ky}\right)\exp\left[ik(x-ct)\right],\\ D_{y}^{*}=-\varepsilon_{11}^{*}kB_{1}^{*}e^{ky}\exp\left[ik(x-ct)\right]. \end{array}\right.$$

### 2.2. Solutions for the Top Piezoelectric Layer H < y < (H + h)

The Love wave solutions in the top piezoelectric layer are

(6) $$\begin{array}{l} w^{\prime }=\left[A_{1}^{\prime }\cos(kb^{\prime }y)+A_{2}^{\prime }\sin(kb^{\prime }y)\right]\exp\left[ik(x-ct)\right],\\ \varphi^{\prime }=\left\{\frac{e_{15}^{\prime }}{\varepsilon_{11}^{\prime }}\left[A_{1}^{\prime }\cos(kb^{\prime }y)+A_{2}^{\prime }\sin(kb^{\prime }y)\right]+\left[B_{1}^{\prime }\cosh(ky)+B_{2}^{\prime }\sinh(ky)\right]\right\}\exp\left[ik(x-ct)\right], \end{array}$$

where $A_{1}^{\prime }$, $A_{2}^{\prime }$, $B_{1}^{\prime }$, and $B_{2}^{\prime }$ are undetermined constants, and $b^{\prime }=\sqrt{{\left(c/c_{layer}\right)}^{2}-1}$. $c_{layer}=\sqrt{{\bar{c}}_{44}^{\prime }/\rho^{\prime }}$ is the bulk shear velocity in the layer with ${\bar{c}}_{44}^{\prime }=c_{44}^{\prime }+\frac{{e_{15}^{\prime }}^{2}}{\varepsilon_{11}^{\prime }}$. The phase velocity of the Love waves satisfies $c_{layer}<c<c_{sub}$, which ensures $b^{*}$ and $b^{\prime }$ are all positive. Finally, the corresponding stress and electric displacement in the top piezoelectric layer are

(7) $$\begin{array}{l} \sigma_{zy}^{\prime }=\left\{kb^{\prime }{\bar{c}}_{44}^{\prime }\left[-A_{1}^{\prime }\sin(kb^{\prime }y)+A_{2}^{\prime }\cos(kb^{\prime }y)\right]+e_{15}^{\prime }k\left[B_{1}^{\prime }\sinh(ky)+B_{2}^{\prime }\cosh(ky)\right]\right\}\exp\left[ik(x-ct)\right],\\ {D^{\prime }}_{y}=-\varepsilon_{11}^{\prime }k\left[B_{1}^{\prime }\sinh(ky)+B_{2}^{\prime }\cosh(ky)\right]\exp\left[ik(x-ct)\right]. \end{array}$$

### 2.3. Solutions for the Inhomogeneous Internal Region 0 < y < H

Sometimes, is assumed that all material coefficients of the middle layer change in the same exponential function variation [38,39], which can provide convenience for decoupling the mechanical components from the electrical field. However, this variation is impossible in actual life. In this contribution, the material variation tendency is not limited, which is the highlight of this paper. No matter what variation form the material parameters of the inhomogeneous layer take along the *y* direction, they can be expressed in the power function theoretically by using Taylor’s series, which means

(8) $$c_{44}=\sum_{n=0}^{\infty }c_{44}^{\left(n\right)}{\left(\frac{y}{H}\right)}^{n}\;,\;e_{15}=\sum_{n=0}^{\infty }e_{15}^{\left(n\right)}{\left(\frac{y}{H}\right)}^{n}\;,\;\varepsilon_{11}=\sum_{n=0}^{\infty }\varepsilon_{11}^{\left(n\right)}{\left(\frac{y}{H}\right)}^{n}\;,\;\rho =\sum_{n=0}^{\infty }\rho^{\left(n\right)}{\left(\frac{y}{H}\right)}^{n}.$$

Theoretically, material coefficients can change in random functions along the thickness direction, which depends on the truncation of the power series and the coefficients $c_{44}^{\left(n\right)}$, $e_{15}^{\left(n\right)}$, $\varepsilon_{11}^{\left(n\right)}$ and $\rho^{\left(n\right)}$. For analysis simplification, the elastic, piezoelectric, and dielectric coefficients as well as the mass density of the layer are all assumed to change linearly in the *y* direction, i.e.,

(9) $$c_{44}=c_{44}^{0}(1-\alpha \frac{y}{H})\;,\;\;e_{15}=e_{15}^{0}(1-\beta \frac{y}{H})\;,\;\varepsilon_{11}=\varepsilon_{11}^{0}(1-\gamma \frac{y}{H})\;,\;\rho =\rho^{0}(1-\eta \frac{y}{H}),$$

where the coefficients *α*, *β*, *γ*, and *η* indicate the profile of the corresponding material gradient along the *y*-axis, and the quantities with superscript “0” are the values of these parameters at *y* = 0. This linear pattern can be used to calculate the effect of the internal stratum induced by internal temperature variations [40], and the investigation of the graded factor on the Love wave propagation properties is of great importance.

The displacement *w* and electrical potential *φ* in the functional graded middle layer 0 < *y* < *H* can be expressed as

(10) $$\left\{\begin{array}{l} w=W(y)\exp\left[ik(x-ct)\right],\\ \varphi =\Phi (y)\exp\left[ik(x-ct)\right]. \end{array}\right.$$

Thus, the governing equations are

(11) $$\begin{array}{l} c_{44}(\frac{d^{2}W}{dy^{2}}-k^{2}W)+e_{15}(\frac{d^{2}\Phi }{dy^{2}}-k^{2}\Phi )+\frac{dc_{44}}{dy}\frac{dW}{dy}+\frac{de_{15}}{dy}\frac{d\Phi }{dy}=-\rho k^{2}c^{2}W,\\ e_{15}(\frac{d^{2}W}{dy^{2}}-k^{2}W)-\varepsilon_{11}(\frac{d^{2}\Phi }{dy^{2}}-k^{2}\Phi )+\frac{de_{15}}{dy}\frac{dW}{dy}-\frac{d\varepsilon_{11}}{dy}\frac{d\Phi }{dy}=0. \end{array}$$

Equation (11) contains two second-order partial differential equations with variable coefficients with respect to *W* and *Φ*. Different from previous works [21,31], an easy numerical solution is developed, which is based on the power function expansion technique along the *y* direction [41,42,43,44], i.e.,

(12) $$W(y)=\sum_{n=0}^{\infty }a_{n}{\left(\frac{y}{H}\right)}^{n}\;,\;\Phi (y)=\sum_{n=0}^{\infty }b_{n}{\left(\frac{y}{H}\right)}^{n}.$$

Substituting Equations (9) and (12) into Equation (11) yields

(13a) $$\begin{array}{l} c_{44}^{0}(1-\alpha \frac{y}{H})\sum_{n=0}^{\infty }\left[(n+2)(n+1)a_{n+2}-{\left(kH\right)}^{2}a_{n}\right]{\left(\frac{y}{H}\right)}^{n}\\ \;\;\;\;\;\;\;\;\;\;+e_{15}^{0}(1-\beta \frac{y}{H})\sum_{n=0}^{\infty }\left[(n+2)(n+1)b_{n+2}-{\left(kH\right)}^{2}b_{n}\right]{\left(\frac{y}{H}\right)}^{n}\\ \;\;\;\;\;\;\;\;\;-\sum_{n=0}^{\infty }(n+1)\left(c_{44}^{0}\alpha a_{n+1}+e_{15}^{0}\beta b_{n+1}\right){\left(\frac{y}{H}\right)}^{n}+\rho^{0}(1-\eta \frac{y}{H}){\left(kH\right)}^{2}c^{2}\sum_{n=0}^{\infty }a_{n}{\left(\frac{y}{H}\right)}^{n}=0\;, \end{array}$$

(13b) $$\begin{array}{l} e_{15}^{0}(1-\beta \frac{y}{H})\sum_{n=0}^{\infty }\left[(n+2)(n+1)a_{n+2}-{\left(kH\right)}^{2}a_{n}\right]{\left(\frac{y}{H}\right)}^{n}\\ \;\;\;\;\;\;\;\;\;\;-\varepsilon_{11}^{0}(1-\gamma \frac{y}{H})\sum_{n=0}^{\infty }\left[(n+2)(n+1)b_{n+2}-{\left(kH\right)}^{2}b_{n}\right]{\left(\frac{y}{H}\right)}^{n}\\ \;\;\;\;\;\;\;\;\;-\sum_{n=0}^{\infty }(n+1)\left(e_{15}^{0}\beta a_{n+1}-\varepsilon_{11}^{0}\gamma b_{n+1}\right){\left(\frac{y}{H}\right)}^{n}=0 \end{array}$$

By equating the terms of ${\left(\frac{y}{H}\right)}^{n}$ in Equation (13) to zero, we can obtain the corresponding recursive relationships of *a_n_* and *b_n_*, i.e.,

$$a_{2}=\frac{1}{2}\left[(\frac{c_{44}^{0}}{{\bar{c}}_{44}^{0}}\alpha +k_{e}^{2}\beta )a_{1}+\frac{e_{15}^{0}}{{\bar{c}}_{44}^{0}}(\beta -\gamma )b_{1}+(1-\frac{c^{2}}{c_{0}^{2}}){\left(kH\right)}^{2}a_{0}\right]\;,$$

(14a) $$b_{2}=\frac{1}{2}\left[\frac{c_{44}^{0}}{{\bar{c}}_{44}^{0}}\frac{e_{15}^{0}}{\varepsilon_{11}^{0}}(\alpha -\beta )a_{1}+(\frac{c_{44}^{0}}{{\bar{c}}_{44}^{0}}\gamma +k_{e}^{2}\beta )b_{1}-\frac{e_{15}^{0}}{\varepsilon_{11}^{0}}\frac{c^{2}}{c_{0}^{2}}{\left(kH\right)}^{2}a_{0}+{\left(kH\right)}^{2}b_{0}\right],$$

$$\begin{array}{l} a_{n+3}=\frac{1}{\left(n+3)(n+2\right)}\left[{\left(n+2\right)}^{2}(\frac{c_{44}^{0}}{{\bar{c}}_{44}^{0}}\alpha +k_{e}^{2}\beta )a_{n+2}+{\left(n+2\right)}^{2}\frac{e_{15}^{0}}{{\bar{c}}_{44}^{0}}(\beta -\gamma )b_{n+2}+(1-\frac{c^{2}}{c_{0}^{2}}){\left(kH\right)}^{2}a_{n+1}\right.\\ \;\;\;\;\;\;\;\;\;\;\left.-(\frac{c_{44}^{0}}{{\bar{c}}_{44}^{0}}\alpha +k_{e}^{2}\beta -\eta \frac{c^{2}}{c_{0}^{2}}){\left(kH\right)}^{2}a_{n}-\frac{e_{15}^{0}}{{\bar{c}}_{44}^{0}}(\beta -\gamma ){\left(kH\right)}^{2}b_{n}\right]\;, \end{array}$$

(14b) $$\begin{array}{l} b_{n+3}=\frac{1}{\left(n+3)(n+2\right)}\left\{{\left(n+2\right)}^{2}\frac{c_{44}^{0}}{{\bar{c}}_{44}^{0}}\frac{e_{15}^{0}}{\varepsilon_{11}^{0}}(\alpha -\beta )a_{n+2}+{\left(n+2\right)}^{2}(\frac{c_{44}^{0}}{{\bar{c}}_{44}^{0}}\gamma +k_{e}^{2}\beta )b_{n+2}-\frac{e_{15}^{0}}{\varepsilon_{11}^{0}}\frac{c^{2}}{c_{0}^{2}}{\left(kH\right)}^{2}a_{n+1}\right.\\ \;\;\;\;\;\;\;\;\;\left.+{\left(kH\right)}^{2}b_{n+1}-\frac{e_{15}^{0}}{\varepsilon_{11}^{0}}\left[\frac{c_{44}^{0}}{{\bar{c}}_{44}^{0}}(\alpha -\beta )-\eta \frac{c^{2}}{c_{0}^{2}}\right]{\left(kH\right)}^{2}a_{n}-(\frac{c_{44}^{0}}{{\bar{c}}_{44}^{0}}\gamma +k_{e}^{2}\beta ){\left(kH\right)}^{2}b_{n}\right\}\;\;,\;\;(n\geq 0) \end{array}$$

Here $k_{e}^{2}=\frac{{e_{15}^{0}}^{2}}{\varepsilon_{11}^{0}{\bar{c}}_{44}^{0}}$, ${\bar{c}}_{44}^{0}=c_{44}^{0}+\frac{{e_{15}^{0}}^{2}}{\varepsilon_{11}^{0}}$, and $c_{0}=\sqrt{{\bar{c}}_{44}^{0}/\rho^{0}}$ are the respectively piezoelectric coupling constant, effective piezoelectric stiffness, and bulk shear wave velocity when the middle layer is homogeneous. *a*_0_, *a*_1_, *b*_0_, *b*_1_ are undetermined coefficients. Once these parameters are calculated, *a_n_* and *b_n_* (*n* > 1) can be obtained using Equation (14). Based on the given solutions, the solutions for the FG piezoelectric middle layer are

(15) $$\left\{\begin{array}{l} w=\sum_{n=0}^{\infty }a_{n}{\left(\frac{y}{H}\right)}^{n}\exp\left[ik(x-ct)\right],\\ \varphi =\sum_{n=0}^{\infty }b_{n}{\left(\frac{y}{H}\right)}^{n}\exp\left[ik(x-ct)\right]. \end{array}\right.$$

Similarly, the stress and electrical displace components are

(16) $$\left\{\begin{array}{l} \sigma_{zy}=\frac{1}{H}\sum_{n=0}^{\infty }(n+1)\left[c_{44}^{0}(1-\alpha \frac{y}{H})a_{n+1}+e_{15}^{0}(1-\beta \frac{y}{H})b_{n+1}\right]{\left(\frac{y}{H}\right)}^{n}\exp\left[ik(x-ct)\right],\\ D_{y}=\frac{1}{H}\sum_{n=0}^{\infty }(n+1)\left[e_{15}^{0}(1-\beta \frac{y}{H})a_{n+1}-\varepsilon_{11}^{0}(1-\gamma \frac{y}{H})b_{n+1}\right]{\left(\frac{y}{H}\right)}^{n}\exp\left[ik(x-ct)\right]. \end{array}\right.$$

### 2.4. The Electric Field in the Air y > (H +h)

Usually, the space above the upper surface of the layer is air. Therefore, the electrical potential function *φ*_0_(*x*, *y*) satisfies the Laplace equation:

(17) $$\nabla^{2}\varphi_{0}(x,y)=0.$$

Given that the electrical potential function in the air satisfies the governing Equation (17), the solutions can be expressed as

(18) $$\left\{\begin{array}{l} \varphi_{0}=F_{0}e^{k\left[(H+h)-y\right]}\exp[ik(x-ct)],\\ D_{y0}=-\varepsilon_{0}kF_{0}e^{k\left[(H+h)-y\right]}\exp[ik(x-ct)], \end{array}\right.$$

where *F*_0_ is also an arbitrary constant, and *ε*_0_ is the dielectric permittivity in the air. It should be stressed that the layer’s surface is directly attached to the air in this paper. When facing a sorption process or surface contamination, Equation (17) is not applicable anymore.

### 2.5. The Phase Velocity Equations

The traction-free surface at *y* = *H* + *h* requires

(19) $$\sigma_{zy}^{\prime }=0.$$

The electrical boundary conditions at *y* = *H* + *h* are

(20a) $$\varphi^{\prime }(x,H+h)=\varphi_{0}(x,H+h)\;,\;D_{y}^{\prime }(x,H+h)=D_{y0}(x,H+h)$$

for the electrically open case and

(20b) $$\varphi^{\prime }(x,H+h)=0$$

for the electrically shorted case.

The continuity conditions at *y* = *H* and *y* = 0 are, respectively,

(21a) $$\left\{\begin{array}{l} \sigma_{zy}^{\prime }(x,H)=\sigma_{zy}(x,H),\;D_{y}^{\prime }(x,H)=D_{y}(x,H),\\ w^{\prime }(x,H)=w(x,H),\;\varphi^{\prime }(x,H)=\varphi (x,H), \end{array}\right.$$

(21b) $$\left\{\begin{array}{l} \sigma_{zy}(x,0)=\sigma_{zy}^{*}(x,0),\;D_{y}(x,0)=D_{y}^{*}(x,0),\\ w(x,0)=w^{*}(x,0),\;\varphi^{*}(x,0)=\varphi (x,0). \end{array}\right.$$

Substituting the displacement and electrical potential expressions in Equations (4), (6), and (15) as well as their corresponding stress and electric displacement components in Equations (4), (7), and (16) into Equations (19)–(21) yields the following linear homogeneous algebraic equations for coefficients $A_{1}^{\prime }$, $A_{2}^{\prime }$, $B_{1}^{\prime }$, $B_{2}^{\prime }$, *a*_0_, *a*_1_, *b*_0_, *b*_1_, $A_{1}^{*}$ and $B_{1}^{*}$:

(22) $$\begin{array}{l} b^{\prime }{\bar{c}}_{44}^{\prime }\left\{-A_{1}^{\prime }\sin\left[kb^{\prime }(H+h)\right]+A_{2}^{\prime }\cos\left[kb^{\prime }(H+h)\right]\right\}\\ \;\;\;\;\;\;\;\;\;\;+e_{15}^{\prime }\left\{B_{1}^{\prime }\sinh\left[k(H+h)\right]+B_{2}^{\prime }\cosh\left[k(H+h)\right]\right\}=0. \end{array}$$

(23a) $$\begin{array}{l} \frac{e_{15}^{\prime }}{\varepsilon_{11}^{\prime }}\left\{A_{1}^{\prime }\cos\left[kb^{\prime }(H+h)\right]+A_{2}^{\prime }\sin\left[kb^{\prime }(H+h)\right]\right\}\\ \;\;\;\;\;\;\;\;\;+\left\{\cosh\left[k(H+h)\right]+\frac{\varepsilon_{11}^{\prime }}{\varepsilon_{0}}\sinh\left[k(H+h)\right]\right\}B_{1}^{\prime }\\ \;\;\;\;\;\;\;\;\;+\left\{\sinh\left[k(H+h)\right]+\frac{\varepsilon_{11}^{\prime }}{\varepsilon_{0}}\cosh\left[k(H+h)\right]\right\}B_{2}^{\prime }=0, \end{array}$$

(23b) $$\begin{array}{l} \frac{e_{15}^{\prime }}{\varepsilon_{11}^{\prime }}\left\{A_{1}^{\prime }\cos\left[kb^{\prime }(H+h)\right]+A_{2}^{\prime }\sin\left[kb^{\prime }(H+h)\right]\right\}\\ \;\;\;\;\;\;\;\;+B_{1}^{\prime }\cosh\left[k(H+h)\right]+B_{2}^{\prime }\sinh\left[k(H+h)\right]=0, \end{array}$$

(24) $$\left\{\begin{array}{l} b^{\prime }{\bar{c}}_{44}^{\prime }\left[-A_{1}^{\prime }\sin(kb^{\prime }H)+A_{2}^{\prime }\cos(kb^{\prime }H)\right]+e_{15}^{\prime }\left[B_{1}^{\prime }\sinh(kH)+B_{2}^{\prime }\cosh(kH)\right]\\ \;\;\;\;\;\;\;\;\;\;\;\;\;\;\;\;\;\;\;\;\;\;\;=\frac{1}{kH}\sum_{n=0}^{\infty }(n+1)\left[c_{44}^{0}(1-\alpha )a_{n+1}+e_{15}^{0}(1-\beta )b_{n+1}\right],\\ -\varepsilon_{11}^{\prime }\left[B_{1}^{\prime }\sinh(kH)+B_{2}^{\prime }\cosh(kH)\right]=\frac{1}{kH}\sum_{n=0}^{\infty }(n+1)\left[e_{15}^{0}(1-\beta )a_{n+1}-\varepsilon_{11}^{0}(1-\gamma )b_{n+1}\right],\\ A_{1}^{\prime }\cos(kb^{\prime }H)+A_{2}^{\prime }\sin(kb^{\prime }H)=\sum_{n=0}^{\infty }a_{n},\\ \frac{e_{15}^{\prime }}{\varepsilon_{11}^{\prime }}\left[A_{1}^{\prime }\cos(kb^{\prime }H)+A_{2}^{\prime }\sin(kb^{\prime }H)\right]+\left[B_{1}^{\prime }\cosh(kH)+B_{2}^{\prime }\sinh(kH)\right]=\sum_{n=0}^{\infty }b_{n},\\ b^{*}{\bar{c}}_{44}^{*}A_{1}^{*}+e_{15}^{*}B_{1}^{*}=\frac{1}{kH}\left(c_{44}^{0}a_{1}+e_{15}^{0}b_{1}\right),\\ -\varepsilon_{11}^{*}B_{1}^{*}=\frac{1}{kH}\left(e_{15}^{0}a_{1}-\varepsilon_{11}^{0}b_{1}\right),\\ A_{1}^{*}=a_{0},\;\\ \frac{e_{15}^{*}}{\varepsilon_{11}^{*}}A_{1}^{*}+B_{1}^{*}=b_{0}. \end{array}\right.$$

Non-trivial solutions can only exist when the determinant of the coefficient matrix of Equations (22), (23a) and (24) is equal to zero, which yields the phase velocity equation of Love wave propagation in this structure for the electrically open case:

(25) $$\left|Q(i,j)\right|=0\;,\;\;(i=1,2,3,\cdots ,10;\;j=1,2,3,\cdots ,10)$$

with

(26) $$\left\{\begin{array}{l} Q(1,1)=-b^{\prime }{\bar{c}}_{44}^{\prime }\sin\left[kb^{\prime }(H+h)\right],\;Q(1,2)=b^{\prime }{\bar{c}}_{44}^{\prime }\cos\left[kb^{\prime }(H+h)\right],\;\;\\ \;\;\;\;\;\;\;\;\;Q(1,3)=e_{15}^{\prime }\sinh\left[k(H+h)\right],\;Q(1,4)=e_{15}^{\prime }\cosh\left[k(H+h)\right],\\ Q(2,1)=\frac{e_{15}^{\prime }}{\varepsilon_{11}^{\prime }}\cos\left[kb^{\prime }(H+h)\right],\;Q(2,2)=\frac{e_{15}^{\prime }}{\varepsilon_{11}^{\prime }}\sin\left[kb^{\prime }(H+h)\right],\\ \;\;\;\;\;\;\;\;\;Q(2,3)=\cosh\left[k(H+h)\right]+\frac{\varepsilon_{11}^{\prime }}{\varepsilon_{0}}\sinh\left[k(H+h)\right],\;\\ \;\;\;\;\;\;\;\;\;Q(2,4)=\sinh\left[k(H+h)\right]+\frac{\varepsilon_{11}^{\prime }}{\varepsilon_{0}}\cosh\left[k(H+h)\right],\\ Q(3,1)=b^{\prime }{\bar{c}}_{44}^{\prime }\sin\left(kb^{\prime }H\right),\;Q(3,2)=-b^{\prime }{\bar{c}}_{44}^{\prime }\cos\left(kb^{\prime }H\right),\;\\ \;\;\;\;\;\;\;Q(3,3)=-e_{15}^{\prime }\sinh\left(kH\right),\;Q(3,4)=-e_{15}^{\prime }\cosh\left(kH\right),\\ \;\;\;\;\;\;\;Q(3,m)=\sum_{n=0}^{\infty }(n+1)\left[c_{44}^{0}(1-\alpha )a_{n+1}+e_{15}^{0}(1-\beta )b_{n+1}\right],\\ Q(4,1)=-\cos\left(kb^{\prime }H\right),\;Q(4,2)=-\sin\left(kb^{\prime }H\right),\;Q(4,m)=\sum_{n=0}^{\infty }a_{n}\\ Q(5,3)=-\varepsilon_{11}^{\prime }\cosh\left(kH\right),\;Q(5,4)=-\varepsilon_{11}^{\prime }\sinh\left(kH\right),\;\\ \;\;\;\;\;\;\;\;Q(5,m)=\frac{1}{kH}\sum_{n=0}^{\infty }(n+1)\left[e_{15}^{0}(1-\beta )a_{n+1}-\varepsilon_{11}^{0}(1-\gamma )b_{n+1}\right],\\ Q(6,1)=-\frac{e_{15}^{\prime }}{\varepsilon_{11}^{\prime }}\cos\left(kb^{\prime }H\right),\;Q(6,2)=-\frac{e_{15}^{\prime }}{\varepsilon_{11}^{\prime }}\sin\left(kb^{\prime }H\right),Q(6,3)=-\cosh\left(kH\right),\\ \;\;\;\;\;\;\;\;Q(6,4)=-\sinh\left(kH\right),\;Q(6,m)=\sum_{n=0}^{\infty }b_{n},\\ Q(7,6)=\frac{1}{kH}c_{44}^{0},\;Q(7,8)=\frac{1}{kH}e_{15}^{0},\;Q(7,9)=-b^{*}{\bar{c}}_{44}^{*},\;Q(7,10)=-e_{15}^{*},\\ Q(8,5)=1,\;Q(8,9)=-1,\\ Q(9,6)=\frac{1}{kH}e_{15}^{0},\;Q(9,8)=-\frac{1}{kH}\varepsilon_{11}^{0},\;Q(9,10)=\varepsilon_{11}^{*},\\ Q(10,7)=1,\;Q(10,9)=-\frac{e_{15}^{*}}{\varepsilon_{11}^{*}},\;Q(10,10)=-1. \end{array}\right.$$

Here m=5, 6, 7, 8, and all the other terms are equal to zero. Similarly, we can obtain the phase velocity equation of the Love wave for the electrically shorted case by using Equations (22), (23b) and (24), which have the same expression as Equation (26) if *ε*_0_ = ∞.

## 3. Numerical Simulations

For revealing the Love wave behavior in the composite structure shown in Figure 1 and graphically showing the effect of the inhomogeneous middle layer, the following material system is considered, i.e., a semi-infinite BaTiO_3_ substrate carrying a PZT4 layer in the middle and a PZT5 layer on the top. The elastic and piezoelectric constants, the mass density and the dielectric constants are summarized in Table 1 [45,46]. For solving Equation (26), we adopt an iterative procedure for the numerical computations. For an initial value of *c*, the determinant of Equation (26) is estimated. A fixed but small increment is added each time until the value of the determinant changes its sign.

### 3.1. Convergence of the Power Series

Firstly, the series convergence is examined. Table 2, Table 3 and Table 4 show the phase velocities of Love waves for some special cases, which mean that 20 terms in the series are sufficient to ensure acceptable accuracy. Additionally, the convergence is closely related to the length *H* of the middle layer and has no relationship with the thickness of the upper layer *h* and the gradient coefficients *α*, *β*, *γ*, and *η*, whether the upper surface is electrically open or shorted. In the following simulation, *kH* is fixed to 1, and the series truncation is chosen as 20.

### 3.2. Verification of the Power Series

For validating the correctness of this method, a special case, i.e., a homogeneous middle layer with *α* = *β* = *γ* = *η* = 0, is considered. Then, the theoretical solutions can easily be derived as

(27) $$\left\{\begin{array}{l} w=\left[A_{0}\cos(kby)+A_{1}\sin(kby)\right]\exp\left[ik(x-ct)\right],\\ \varphi =\left\{\frac{e_{15}^{0}}{\varepsilon_{11}^{0}}\left[A_{0}\cos(kby)+A_{1}\sin(kby)\right]+\left[B_{0}\cosh(ky)+B_{1}\sinh(ky)\right]\right\}\exp\left[ik(x-ct)\right],\\ \sigma_{zy}=\left\{{\bar{c}}_{44}^{0}kb\left[-A_{0}\sin(kby)+A_{1}\cos(kby)\right]+e_{15}^{0}k\left[B_{0}\sinh(ky)+B_{1}\cosh(ky)\right]\right\}\exp\left[ik(x-ct)\right],\\ D_{y}=-\varepsilon_{11}^{0}k\left[B_{0}\sinh(ky)+B_{1}\cosh(ky)\right]\exp\left[ik(x-ct)\right], \end{array}\right.$$

with $b=\sqrt{{\left(c/c_{0}\right)}^{2}-1}$, and *A*_0_, *A*_1_, *B*_0_, and *B*_1_ being undetermined coefficients. Here, it is assumed that the velocity of Love waves is larger than the bulk velocity of the homogeneous middle layer, i.e., *c* > *c*_0_. If *c* < *c*_0_, the sine and cosine functions in Equation (27) can be changed to hyperbolic sine and cosine functions, respectively. Similarly, substituting Equations (4)–(7) and (27) into the continuity condition in Equation (21) yields

(28) $$\left\{\begin{array}{l} b^{\prime }{\bar{c}}_{44}^{\prime }\left[-A_{1}^{\prime }\sin(kb^{\prime }H)+A_{2}^{\prime }\cos(kb^{\prime }H)\right]+e_{15}^{\prime }\left[B_{1}^{\prime }\sinh(kH)+B_{2}^{\prime }\cosh(kH)\right]\\ \;\;\;\;\;\;\;\;\;\;\;\;\;\;\;\;\;={\bar{c}}_{44}^{0}b\left[-A_{0}\sin(kbH)+A_{1}\cos(kbH)\right]+e_{15}^{0}\left[B_{0}\sinh(kH)+B_{1}\cosh(kH)\right],\\ -\varepsilon_{11}^{\prime }\left[B_{1}^{\prime }\sinh(kH)+B_{2}^{\prime }\cosh(kH)\right]=-\varepsilon_{11}^{0}\left[B_{0}\sinh(kH)+B_{1}\cosh(kH)\right],\\ A_{1}^{\prime }\cos(kb^{\prime }H)+A_{2}^{\prime }\sin(kb^{\prime }H)=A_{0}\cos(kbH)+A_{1}\sin(kbH),\\ \frac{e_{15}^{\prime }}{\varepsilon_{11}^{\prime }}\left[A_{1}^{\prime }\cos(kb^{\prime }H)+A_{2}^{\prime }\sin(kb^{\prime }H)\right]+\left[B_{1}^{\prime }\cosh(kH)+B_{2}^{\prime }\sinh(kH)\right]\\ \;\;\;\;\;\;\;\;\;\;\;\;\;\;\;\;\;=\frac{e_{15}^{0}}{\varepsilon_{11}^{0}}\left[A_{0}\cos(kbH)+A_{1}\sin(kbH)\right]+\left[B_{0}\cosh(kH)+B_{1}\sinh(kH)\right],\\ b^{*}{\bar{c}}_{44}^{*}A_{1}^{*}+e_{15}^{*}B_{1}^{*}={\bar{c}}_{44}^{0}bA_{1}+e_{15}^{0}B_{1},\\ -\varepsilon_{11}^{*}B_{1}^{*}=-\varepsilon_{11}^{0}B_{1},\\ A_{1}^{*}=A_{0},\;\\ \frac{e_{15}^{*}}{\varepsilon_{11}^{*}}A_{1}^{*}+B_{1}^{*}=\frac{e_{15}^{0}}{\varepsilon_{11}^{0}}A_{0}+B_{0}. \end{array}\right.$$

Equations (22), (23a) and (28) totally contain ten linear homogeneous algebraic equations with ten undetermined constants for the electrically open case. Non-trivial solutions can exist only when the determinant of the coefficient matrix is equal to zero, i.e.,

(29) $$\left|P(i,j)\right|=0\;,\;\;(i=1,2,3,\cdots ,10;\;j=1,2,3,\cdots ,10)$$

with

(30) $$\left\{\begin{array}{l} P(1,1)=-b^{\prime }{\bar{c}}_{44}^{\prime }\sin\left[kb^{\prime }(H+h)\right],\;P(1,2)=b^{\prime }{\bar{c}}_{44}^{\prime }\cos\left[kb^{\prime }(H+h)\right],\;\;\\ \;\;\;\;\;\;P(1,3)=e_{15}^{\prime }\sinh\left[k(H+h)\right],\;P(1,4)=e_{15}^{\prime }\cosh\left[k(H+h)\right],\\ P(2,1)=\frac{e_{15}^{\prime }}{\varepsilon_{11}^{\prime }}\cos\left[kb^{\prime }(H+h)\right],\;P(2,2)=\frac{e_{15}^{\prime }}{\varepsilon_{11}^{\prime }}\sin\left[kb^{\prime }(H+h)\right],\\ \;\;\;\;\;P(2,3)=\cosh\left[k(H+h)\right]+\frac{\varepsilon_{11}^{\prime }}{\varepsilon_{0}}\sinh\left[k(H+h)\right],\;\\ \;\;\;\;\;P(2,4)=\sinh\left[k(H+h)\right]+\frac{\varepsilon_{11}^{\prime }}{\varepsilon_{0}}\cosh\left[k(H+h)\right],\\ P(3,1)=b^{\prime }{\bar{c}}_{44}^{\prime }\sin\left(kb^{\prime }H\right),\;P(3,2)=-b^{\prime }{\bar{c}}_{44}^{\prime }\cos\left(kb^{\prime }H\right),\;\\ \;\;\;\;P(3,3)=-e_{15}^{\prime }\sinh\left(kH\right),\;P(3,4)=-e_{15}^{\prime }\cosh\left(kH\right),\\ \;\;\;\;P(3,5)=-{\bar{c}}_{44}^{0}b\sin\left(kbH\right)-\frac{{e_{15}^{0}}^{2}}{\varepsilon_{11}^{0}}\sinh\left(kH\right),\;P(3,6)=\frac{1}{kH}\left[{\bar{c}}_{44}^{0}\cos\left(kbH\right)-\frac{{e_{15}^{0}}^{2}}{\varepsilon_{11}^{0}}\cosh\left(kH\right)\right],\\ \;\;\;P(3,7)=e_{15}^{0}\sinh\left(kH\right),\;P(3,8)=\frac{1}{kH}e_{15}^{0}\cosh\left(kH\right),\;\;\\ P(4,1)=-\cos\left(kb^{\prime }H\right),\;P(4,2)=-\sin\left(kb^{\prime }H\right),\;P(4,5)=\cos\left(kbH\right),P(4,6)=\sin\left(kbH\right),\;\\ P(5,3)=-\varepsilon_{11}^{\prime }\cosh\left(kH\right),\;P(5,4)=-\varepsilon_{11}^{\prime }\sinh\left(kH\right),\;P(5,5)=e_{15}^{0}\sinh\left(kH\right),\;\\ \;\;\;\;P(5,6)=\frac{1}{kH}e_{15}^{0}\cosh\left(kH\right),\;P(5,7)=-\varepsilon_{11}^{0}\sinh\left(kH\right),\;P(5,8)=-\frac{1}{kH}\varepsilon_{11}^{0}\cosh\left(kH\right),\;\\ P(6,1)=-\frac{e_{15}^{\prime }}{\varepsilon_{11}^{\prime }}\cos\left(kb^{\prime }H\right),\;P(6,2)=-\frac{e_{15}^{\prime }}{\varepsilon_{11}^{\prime }}\sin\left(kb^{\prime }H\right),P(6,3)=-\cosh\left(kH\right),\\ \;\;\;\;\;P(6,4)=-\sinh\left(kH\right),\;P(6,5)=\frac{e_{15}^{0}}{\varepsilon_{11}^{0}}\left[\cos\left(kbH\right)-\cosh\left(kH\right)\right],\\ \;\;\;\;P(6,6)=\frac{1}{kH}\frac{e_{15}^{0}}{\varepsilon_{11}^{0}}\left[\frac{1}{b}\sin\left(kbH\right)-\sinh\left(kH\right)\right],\;P(6,7)=\cosh\left(kH\right),\;P(6,8)=\frac{1}{kH}\sinh\left(kH\right),\\ P(7,6)=\frac{1}{kH}c_{44}^{0},\;P(7,8)=\frac{1}{kH}e_{15}^{0},\;P(7,9)=-b^{*}{\bar{c}}_{44}^{*},\;P(7,10)=-e_{15}^{*},\\ P(8,5)=1,\;P(8,9)=-1,\\ P(9,6)=\frac{1}{kH}e_{15}^{0},\;P(9,8)=-\frac{1}{kH}\varepsilon_{11}^{0},\;P(9,10)=\varepsilon_{11}^{*},\\ P(10,7)=1,\;P(10,9)=-\frac{e_{15}^{*}}{\varepsilon_{11}^{*}},\;P(10,10)=-1. \end{array}\right.$$

All other components of *P*(*i*,*j*) are equal to zero. Comparing Equation (30) with Equation (26), we find that ***P*** and ***Q*** have the same expression, except for *P*(*i*,*j*) and *Q*(*i*,*j*) (*i* = 3, 4, 5, 6; *j* = 5, 6, 7, 8). Table 5 and Table 6 respectively provide the values of *P*(*i*,*j*) and *Q*(*i*,*j*) when *kH* = 1, *kh* = 10 and *c* = 2666.9 m/s, in which *P*(*i*,*j*) is calculated using the theoretical solution, and *Q*(*i*,*j*) is obtained using the power function expansion method. It can be seen that *P*(*i*,*j*) and *Q*(*i*,*j*) are almost the same, which indicates that the power series has high precision and can be used to solve Love waves in this composite structure.

### 3.3. The Effect of the FG Piezoelectric Layer

**Figure 2:**
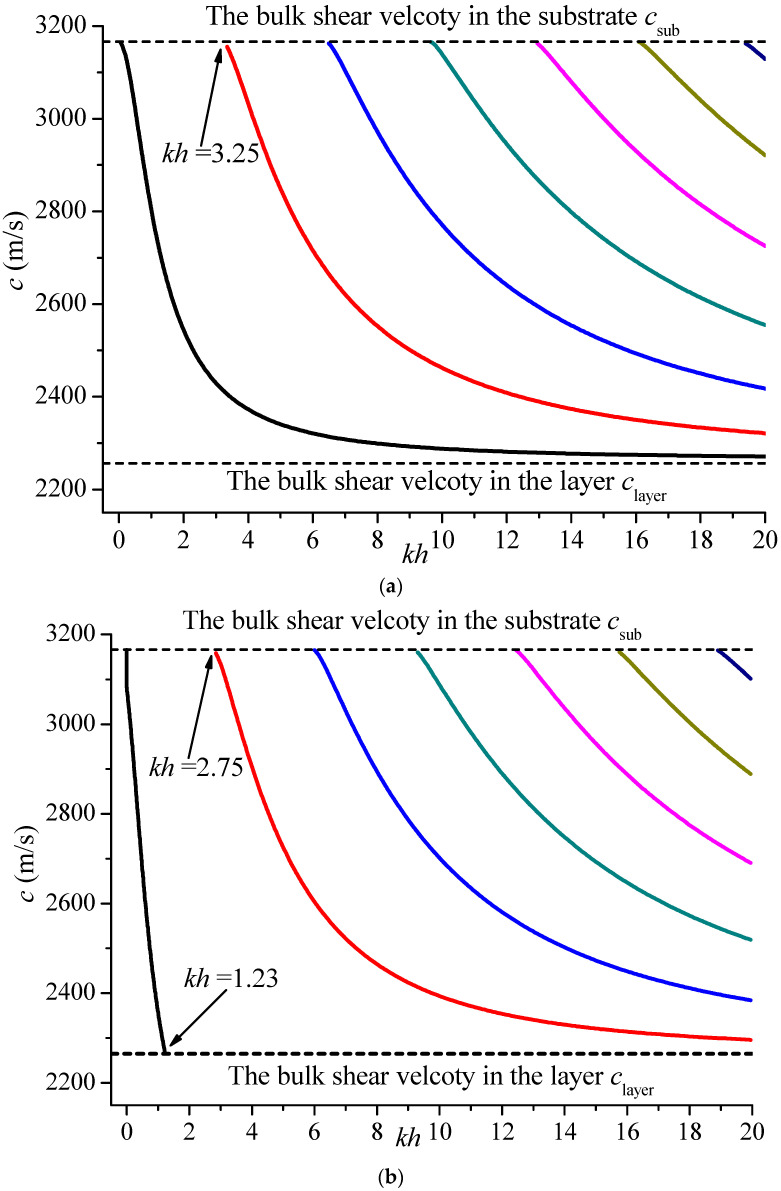
Dispersion curves of Love waves in the PZT5/BaTiO_3_ system (*H* = 0). (**a**) electrically open case; (**b**) electrically shorted case.

After validating the series, this method is adopted to solve the Love waves, and the effect of the FG piezoelectric layer is systematically investigated. Firstly, the dispersion curves of Love waves in the PZT5/BaTiO_3_ system without a middle layer, i.e., *H* = 0, is calculated and shown in Figure 2. For the electrically open case, the phase velocities of all the modes initiate at the bulk shear wave velocity of the half-space medium and approach the bulk shear wave velocity of the piezoelectric layer, and the higher modes appear periodically as the non-dimensional number *kh* is increased, which validates the correctness of the present solutions to some extent [19,41]. However, for the electrically shorted case in Figure 2b, the fundamental mode (0th mode) exists only for some certain non-dimensional wave numbers. When *kh* > 1.23, the velocity is smaller than the bulk shear wave velocity of PZT5, and this mode has changed into the B-G wave. A similar phenomenon was revealed and proved previously by Liu et al. [47] and Li et al. [48], which will not be explored here.

**Figure 3:**
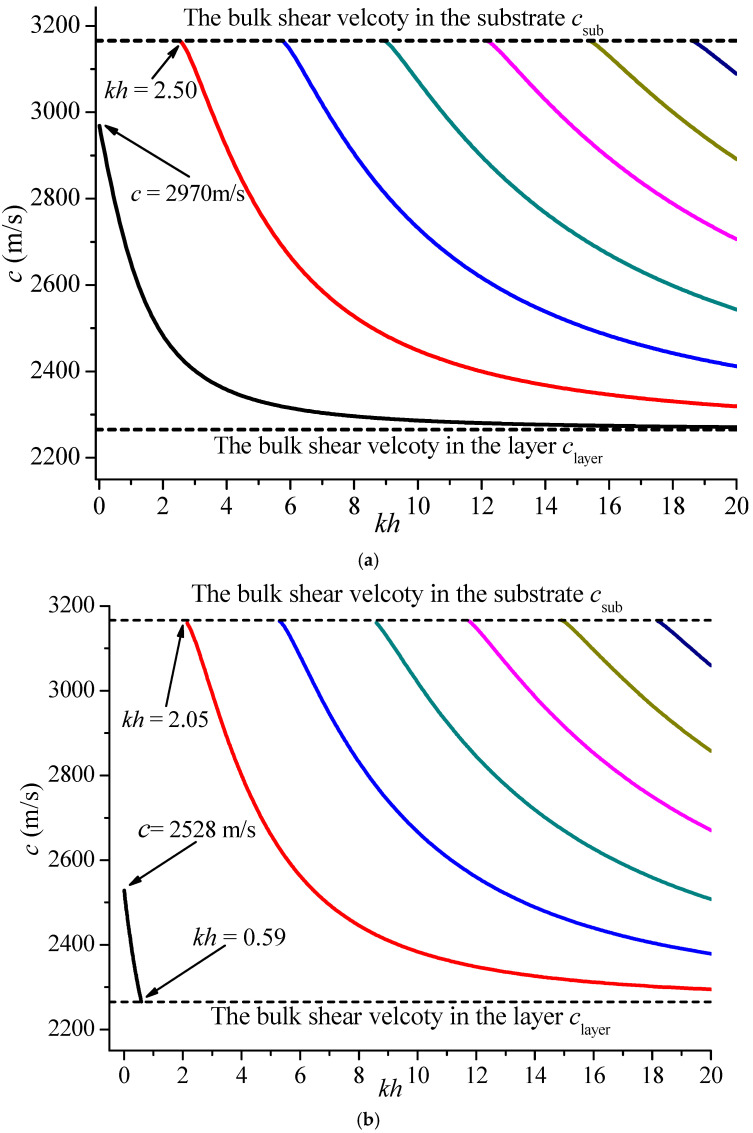
Dispersion curves of Love waves in the PZT5/BaTiO_3_ system with a homogeneous PZT4 middle layer when *kH* = 1. (**a**) electrically open case; (**b**) electrically shorted case.

Figure 3 illustrates the dispersion curves of Love waves in the PZT5/BaTiO_3_ system with a homogeneous PZT4 middle layer for electrically open and shorted cases when *kH* = 1. It is seen that the phase velocities of all higher modes initiate at the bulk shear wave velocity of the half-space medium and approach the bulk shear wave velocity of PZT5 as the non-dimensional number *kh* is increased, which is the same as Figure 2. However, the middle layer PZT4 has reduced the initial value of the fundamental mode for the electrically open case. It is 2790 m/s, which is smaller than the bulk velocity of the substrate. For the electrically shorted case, this mode appears only when the non-dimensional wave number *kh* belongs to (0, 0.59), which means the middle layer lowers the initial phase velocity value and shrinks its existing region.

In addition, the corresponding critical values of the non-dimensional wave number are at the new higher mode appearing in the electrically open and shorted case are different. Taking the first mode, for instance, i.e., the red curve, *kh* = 2.05 for the electrically shorted case, and this mode is postponed until *kh* = 2.50 for the electrically open case. Compared with Figure 2, it can be concluded that increasing the middle layer makes higher modes appear earlier.

**Figure 4:**
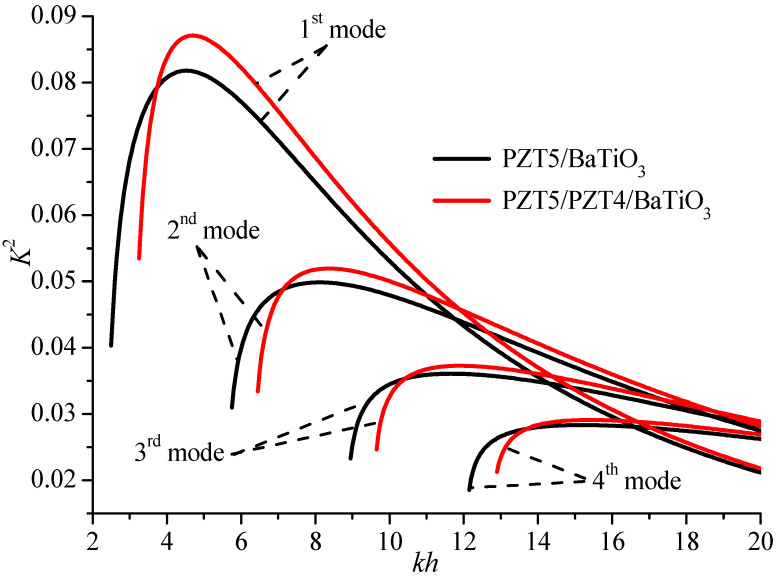
The electromechanical coupling factor of the first four higher modes with and without the middle layer (*kH* = 1).

For SAW devices, the electromechanical coupling factor *K*^2^ is defined as follows [1,19]:

(31) $$K^{2}=\frac{2(c_{\mathrm{open}}-c_{\mathrm{shorted}})}{c_{\mathrm{open}}},$$

where *c*_open_ and *c*_shorted_ are the phase velocities for the electrically open and shorted cases, respectively. *K*^2^ is an important material parameter for the design of SAW devices and sensors because it is directly related to the efficiency of a transducer in converting electrical energy to mechanical energy and vice versa. Figure 4 gives the *K*^2^ of first four higher modes in the composite structure with or without the PZT4 middle layer. The implementation of the PZT4 layer increases the electromechanical coupling factors of all modes, which clearly improves the Love wave performance. The first mode in Figure 4 has the largest peak value of *K*^2^, which should be chosen firstly for surface acoustic wave devices. Additionally, the ratio between the upper layer and the wavelength (or the *kH* value) is also adopted appropriately, so that *K*^2^ can be chosen as the maximum.

**Figure 5:**
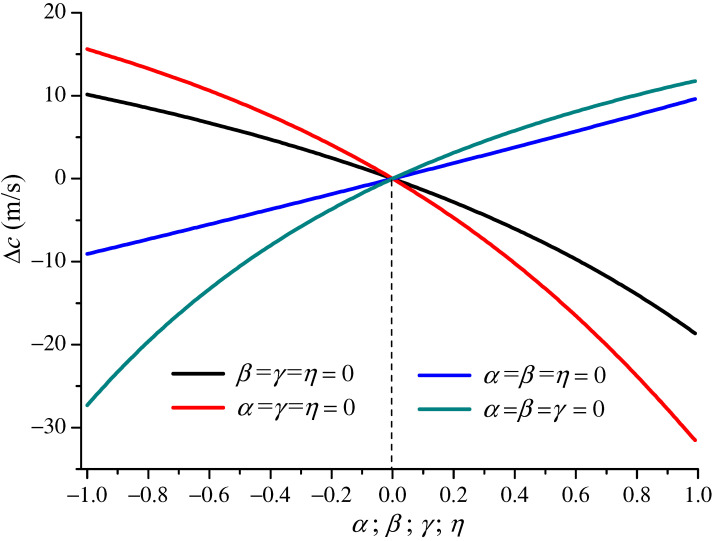
The phase velocity shifts of the first mode caused by different graded parameters of the middle layer when *kh* = 10 and *kH* = 1.

For quantitatively revealing the effect of the graded parameter on wave propagation, the phase velocity shift ∆*c* is defined as ∆*c* = *c* − $\bar{c}$, where *c* is the wave speed when one material parameter changes, and $\bar{c}$ represents the speed of Love waves with the non-graded middle layer. Meanwhile, in order to compare the influence of the elastic material, piezoelectric coefficient, dielectric permittivity, and mass density, only one parameter changes during the analyzing process. For example, the piezoelectric coefficient, dielectric permittivity, and mass density of the middle layer are kept at zero, i.e., *β* = *γ* = *η* = 0, when we focus on the effect of the elastic coefficient, and the same method is used for other parameters. Figure 5 presents the phase velocity shift Δ*c* of the first mode caused by the non-uniform distribution of material properties for the electrically shorted case when *kh* = 10. The curves of phase velocity shift in the electrically open case have similar tendencies as shown in Figure 5, which are not depicted here for the purpose of simplification. It can be seen from Figure 5 that the increase in elastic and piezoelectric parameters, i.e., *α* and *β*, decelerates the Love waves, whereas the increase in dielectric permittivity and mass density i.e., *γ* and *η*, hastens the propagation. The decreasing *α* and *β* or increasing *γ* and *η* enhances the stiffness of the whole structure and then hastens the Love wave propagation. Additionally, the effect of the piezoelectric coefficient is comparable with that of the elastic constant. The absolute value of the speed shift attributed to the change in mass density is larger than that attributed to dielectric permittivity. These tendencies can be used to distinguish the effects of graded coefficients on the Love wave characteristics in composite structures with FGM.

## 4. The Potential Engineering Application

For piezoelectric wafers, the corrosive environment or damage under periodic mechanical or thermal loading usually result in material damage. Under this condition, a small flaw or crack may exist during device production, or the piezoelectric damage phenomenon occurs near the bonding interface between the substrate and the top layer. Given that such a flaw or crack has faint effects, this kind of piezoelectric damage does not affect the elastic, dielectric, and density properties of materials. However, the piezoelectric coefficient may change, which makes the substrate or upper sensitive layer inhomogeneous. In the following section, we investigate the effect of the FG piezoelectric layer that is caused by piezoelectric damage occurring at the interface, i.e., *α* = *γ = η* = 0 and *β* ≠ 0. Cases 1 and 2 in the present contribution are for two different interfaces due to piezoelectric damage. Case 1 corresponds to the substrate piezoelectric damage, i.e., $c_{44}^{0}=c_{44}^{*}$, $e_{15}^{0}=e_{15}^{*}$, $\varepsilon_{11}^{0}=\varepsilon_{11}^{*}$, and  $\rho^{0}=\rho^{*}$. Case 2 corresponds to the upper piezoelectric layer damage, i.e., $c_{44}^{0}=c_{44}^{\prime }$, $e_{15}^{0}=e_{15}^{\prime }$, $\varepsilon_{11}^{0}=\varepsilon_{11}^{\prime }$, and $\rho^{0}=\rho^{\prime }$. Here, the parameter *β* in both cases is considered as the piezoelectric damage parameter.

**Figure 6:**
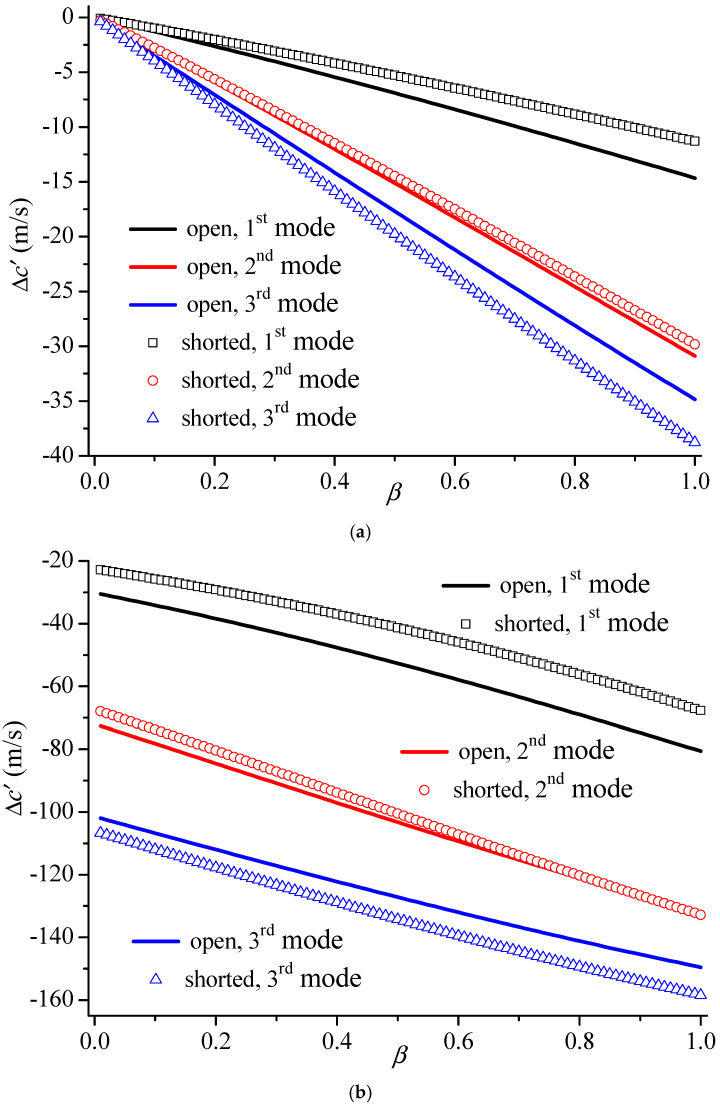
The phase velocity shifts of the first three higher modes versus the piezoelectric damage parameter *β* when *kh* = 10 and *kH* = 1. (**a**) Case 1; (**b**) Case 2.

Similarly to Figure 5, the phase velocity shift $∆c^{\prime }=c-c^{\prime }$ is also defined, in which *c* is the speed of Love waves when piezoelectric parameter *β* changes, and $c^{\prime }$ represents the speed without the middle layer, i.e., the PZT5 layer perfectly bonded on the BaTiO_3_ substrate. Figure 6 demonstrates the phase velocity shifts of the first three higher modes with the damage parameter *β* when *kh* = 10 and *kH* = 1. By comparing Figure 6a,b, it can be concluded that the damage effect of the upper piezoelectric layer is more evident than that of the piezoelectric substrate. Firstly, the phase velocity shift in Figure 6b is larger than that in Figure 6a. Secondly, all curves initiate zero when damage parameter *β* equals zero for Case 1. However, these initial values are not null under the same condition for Case 2, as the thickness of the middle layer is not zero when *β* = 0. Meanwhile, the bulk shear velocity in the piezoelectric layer is smaller than that of substrate.

**Figure 7:**
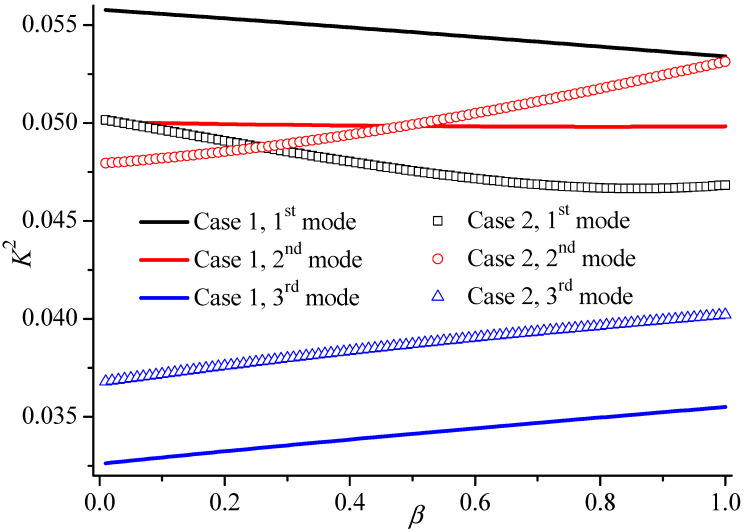
The electromechanical coupling factor of the first three higher modes versus the piezoelectric damage parameter *β* when *kh* = 10 and *kH* = 1.

At same time, the *K*^2^ variation of the first three higher modes versus the piezoelectric damage parameter *β* is illustrated in Figure 7. The piezoelectric damage lowers the electromechanical coupling factor of the first mode. However, with the increasing *β*, the *K*^2^ of the third mode increases, which means the energy conversion rate has been improved by the piezoelectric damage. That is, the *K*^2^ of the second mode can be larger than that of first mode when the piezoelectric coefficient of the upper layer decreases, which may provide a potential design method for sensor applications.

If the upper layer and the substrate have been bonded for a long time, the material near the bonding interface must be influenced by the two different media. Then, a non-uniform transition buffer layer exists, with its material properties changing smoothly. In addition, from the perspective of material manufacturing, an internal stratum can sometimes be designed as a buffer layer to avoid the stress discontinuity of the interface. Therefore, we considered it as the third case, with the material parameters changing from the substrate to the upper layer smoothly, i.e., $c_{44}^{0}=c_{44}^{*}$, $e_{15}^{0}=e_{15}^{*}$, $\varepsilon_{11}^{0}=\varepsilon_{11}^{*}$, $\rho^{0}=\rho^{*}$, $c_{44}^{0}(1-\alpha )=c_{44}^{\prime }$, $e_{15}^{0}(1-\beta )=e_{15}^{\prime }$, $\varepsilon_{11}^{0}(1-\gamma )=\varepsilon_{11}^{\prime }$, and $\rho^{0}(1-\eta )=\rho^{\prime }$. For the BaTiO_3_ substrate and PZT5 layer in this text, *α* = 0.5914, *β* = −0.0789, *γ* = 0.1741, and *η* = −0.3596.

**Figure 8:**
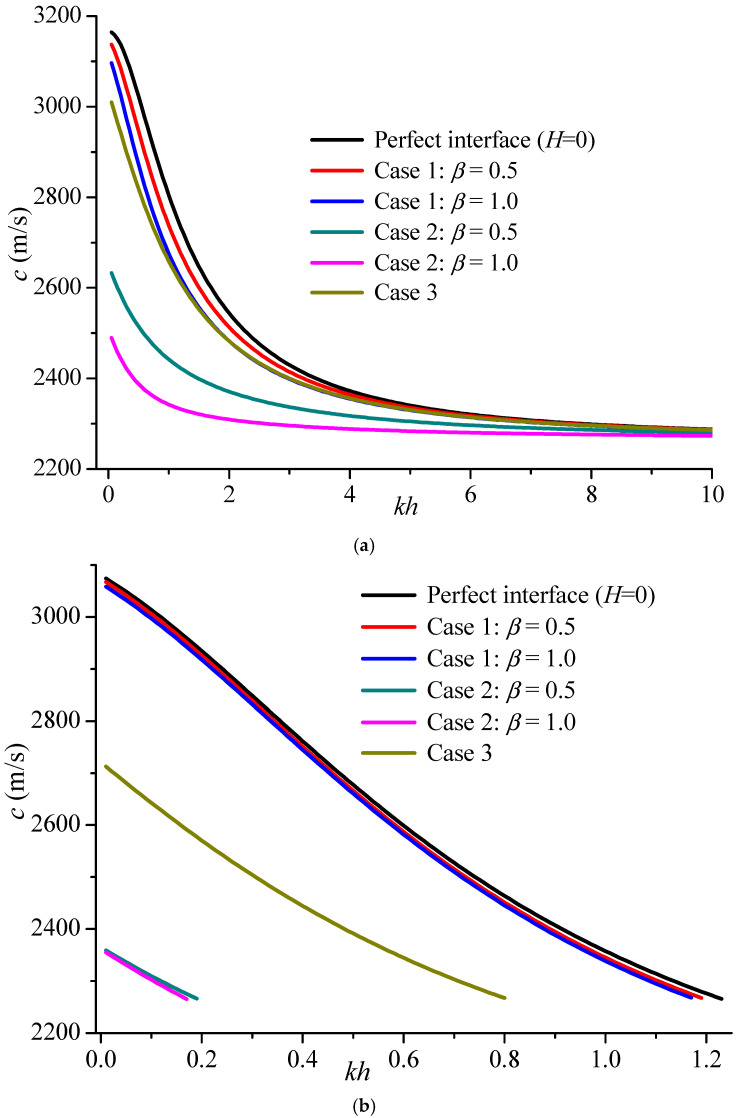
The phase velocity comparison of several cases on the fundamental mode (*kh* = 10). (**a**) electrically open case; (**b**) electrically shorted case.

Figure 8 gives the phase velocity comparison of the fundamental mode for the three kinds of cases above when the upper surface is electrically open and shorted. Case 2 has a greater effect on Love propagation than Case 1, and the effect of Case 3 is between them. For the electrically open surface, the three cases decrease the initial value of the fundamental mode. However, for the electrically shorted case, the three kinds of cases not only decrease the initial value but also shrink the existing region.

**Figure 9:**
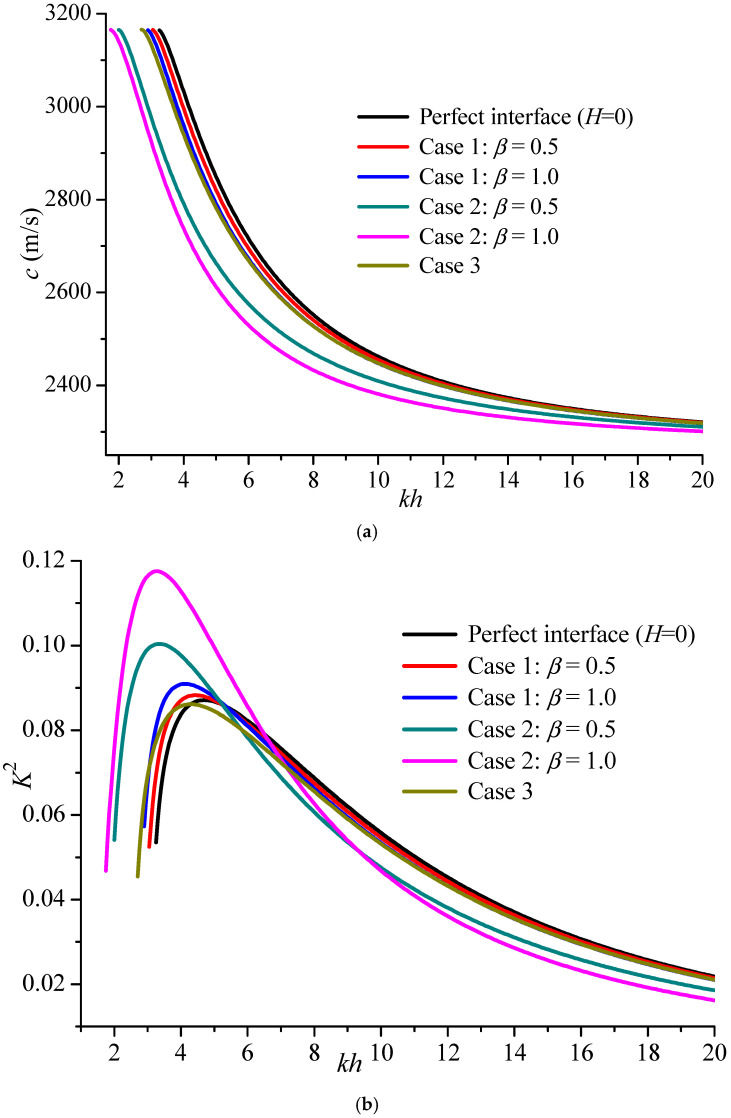
The phase velocity and electromechanical coupling factor comparisons of cases for the first mode (*kh* = 10). (**a**) The phase velocity comparison of three cases; (**b**) The *K*^2^ comparison.

The variation tendency of the first Love mode with an electrically open surface is considered in Figure 9a. Different from the effect on the fundamental mode, the piezoelectric damage in the substrate or upper layer and transition FG internal layer do not change the initial value. However, they all make new Love modes appear earlier as compared with the PZT5A/BaTiO_3_ composite structure, and a larger *β* value has a more evident effect on the wave propagation. Furthermore, the *K*^2^ comparison of the three cases for the first mode is shown in Figure 9b, in which *β* = 1 for the upper layer piezoelectric damage has the largest peak value. The piezoelectric coefficient decreases because of the piezoelectric damage. On the contrary, the energy conversion rate has been improved, which means the emergence of these imperfections is not deleterious for sensor applications.

**Figure 10:**
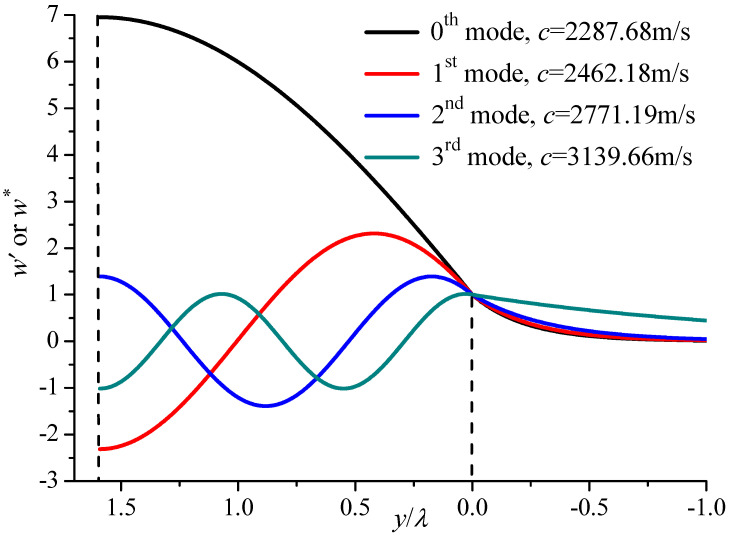
The relative displacement contributions of the first four modes along the thickness direction in the PZT5/BaTiO_3_ substrate when the upper surface is electrically open (*kh* = 10).

The displacement distribution is also an important performance index for SAW devices. Firstly, the relative displacement component of the first four modes along the thickness direction in the PZT5/BaTiO_3_ substrate is calculated and shown in Figure 10. In this paper, the values of the displacement and stress components have been normalized in such a way that the displacement at the interface between the upper layer and substrate is equal to one, i.e., $A_{1}^{*}=1$ at *y* = 0. The fundamental mode has no nodal points, and the first, second and third modes have one, two and three nodal points along the thickness direction. The higher mode has smaller vibration amplitude compared with the fundamental mode, which also can be seen from Figure 10. At the BaTiO_3_ substrate, the displacement attenuates rapidly and approaches zero, which shows the benefits of SAW devices, as most of the energy is concentrated in the upper layer.

**Figure 11:**
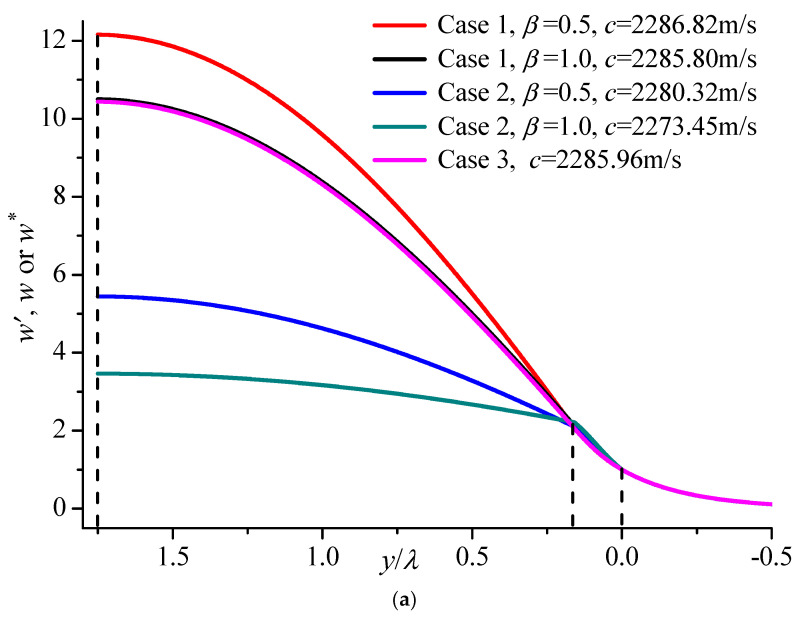

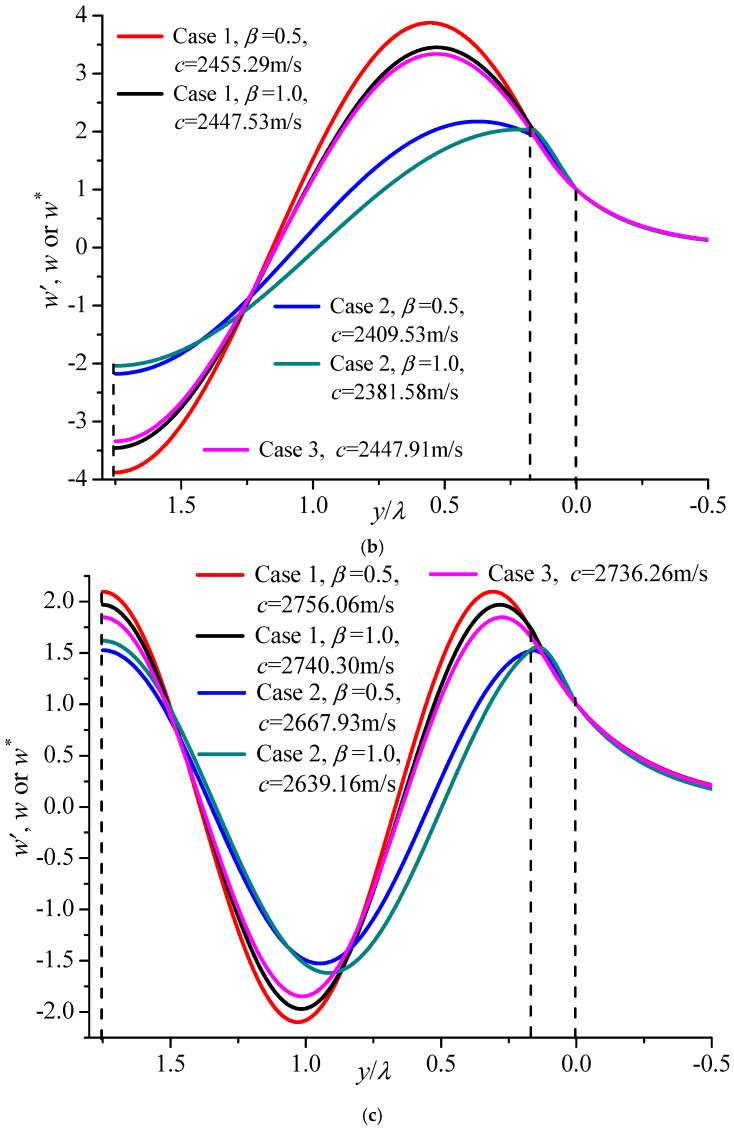

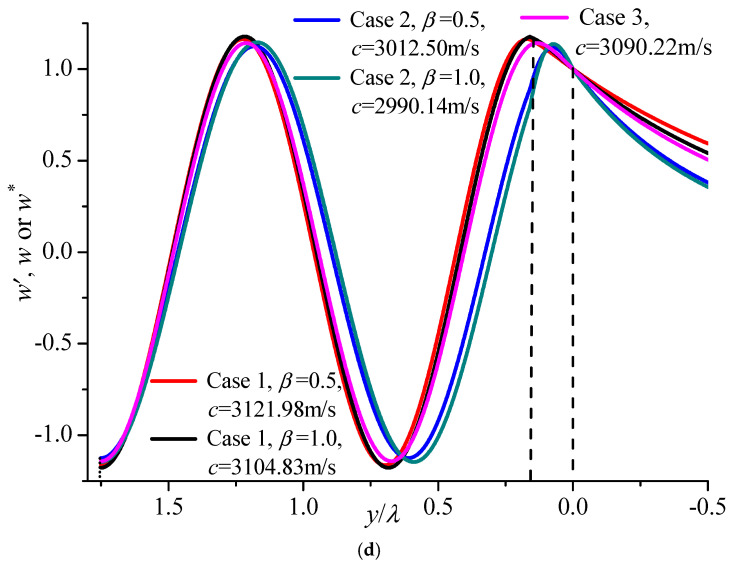
The relative displacement contribution comparison of three cases with the upper surface electrically open (*kh* = 10). (**a**) The fundamental mode; (**b**) The first mode; (**c**) The second mode; (**d**) The third mode.

The relative displacement contributions for the three cases above are shown in Figure 11, in which the region in the dashed lines represents the internal layer. The effect of Case 3 is similar to Case 1 when *β* = 1, such as is shown in Figure 8 and Figure 9a. The phase velocities of these two cases are approximately equal, which causes the displacement distributions to almost overlap. It can be seen From Figure 11a that the displacement of the fundamental Love mode deflects because of the imperfection cases specified above. Overall, the effect of piezoelectric damages in the substrate and upper layer and the transition FG piezoelectric layer for higher modes is tiny, which can be seen from the third mode in Figure 11c,d. In particular, the effect displacement in the BaTiO_3_ substrate changes little, which is because the displacement in the half-space decreases to zero along the thickness direction.

## 5. Conclusions

In summary, the power-series technique was used to solve the Love wave propagation in a composite structure, which consists of a piezoelectric substrate, a sensitive upper piezoelectric layer, and an inhomogeneous internal stratum of finite depth. The good convergence and high precision of this method were illustrated. Some numerical examples are provided to illustrate the detailed effect of material inhomogeneity on the phase velocities and the displacement components, which yields the following conclusions.

(1) The middle layer significantly affects the characteristics of the fundamental Love wave mode, not only in terms of the initial value of the phase velocity, but also the existing region for the electrically shorted condition.
(2) For PZT 5A/BaTiO_3_ composites, the first mode has the largest peak value of the electromechanical coupling factor compared with higher modes, and the additional middle layer can increase the energy efficiency, which improves the Love wave performance evidently.
(3) For the inhomogeneous internal stratum, the Love waves propagate slowly with the reduction of the elastic and piezoelectric coefficients. Moreover, the effects of the dielectric permittivity and the mass density are opposite.
(4) The appearance of substrate and upper layer piezoelectric damages or a functional graded transition layer can significantly affect the Love wave propagation, including the value of the phase velocity, the existing region of the fundamental mode, the electro-mechanical coupling factor, and the displaced contribution.

As the final remark, the power-series expansion method applied in this paper could be also used in the study of acoustic waves along other analogous systems with inhomogeneous materials.

## Figures and Tables

**Table 1:** Material parameters.

Material	$c_{44}\;(\mathrm{Gpa})$	$\rho \;(\mathrm{kg}/m^{3})$	$e\;(C/m^{2})$	$\varepsilon_{11}\;(10^{-9}F/m)$	Bulk Velocity
PZT5	21.1	7750	12.3	8.11	*C*_layer_ = 2265 m/s
PZT4	25.6	7500	12.7	6.46	*C*_middle_ = 2597 m/s
BaTiO_3_	43.9	5700	11.4	9.82	*c*_sub_ = 3166 m/s

**Table 2:** Love wave velocities (m/s) for the homogeneous middle layer (*α* = *β* = *γ* = *η* = 0) using the power function expansion method (*kh* = 10, and *kH* = 1).

*n*	6	8	10	12	15
Open case	2286.190 2448.515 2732.260 3073.151	2286.190 2448.516 2732.262 3073.152	2286.190 2448.516 2732.262 3073.152	2286.190 2448.516 2732.262 3073.152	2286.190 2448.516 2732.262 3073.152
Shorted case	2383.603 2666.857 3020.240	2383.603 2666.858 3020.240	2383.603 2666.859 3020.240	2383.603 2666.859 3020.240	2383.603 2666.859 3020.240

**Table 3:** Love wave velocities (m/s) for different graded parameters using the power function expansion method when the upper surface is electrically shorted (*kH* = 1).

*n*	6	8	10	12	15
*kh* = 5, *α* = *β* = *γ* = *η* = 0	2660.739	2660.742	2660.742	2660.742	2660.742
*kh* = 10, *α* = *β* = *γ* = *η* = 0	2383.603 2666.857 3020.240	2383.603 2666.858 3020.240	2383.603 2666.859 3020.240	2383.603 2666.859 3020.240	2383.603 2666.859 3020.240
*kh* = 10, *α* = 0.5, *β* = *γ* = *η* = 0	2375.834 2647.774 2999.159	2375.795 2647.780 2999.431	2375.794 2647.785 2999.443	2375.794 2647.786 2999.443	2375.794 2647.786 2999.443
*kh* = 10, *β* = 0.5, *α* = *γ* = *η* = 0	2370.051 2634.974 2986.694	2370.299 2636.269 2989.223	2370.351 2636.354 2989.090	2370.345 2636.325 2989.041	2370.344 2636.324 2989.045
*kh* = 10, *γ* = 0.5, *α* = *β* = *η* = 0	2388.314 2679.159 3034.118	2388.347 2679.302 3034.454	2388.351 2679.316 3034.472	2388.351 2679.317 3034.474	2388.351 2679.317 3034.474
*kh* = 10, *η* = 0.5, *α* = *β* = *γ* = 0	2390.546 2693.603 3072.490	2390.586 2693.743 3072.748	2390.587 2693.750 3072.774	2390.587 2693.750 3072.773	2390.587 2693.750 3072.773

**Table 4:** Love wave velocities (m/s) for different thicknesses of middle layers using the power function expansion method when the upper surface is electrically shorted (*kh* = 10, and *α* = *β* = *γ* = *η* = 0).

*n*	6	8	10	12	15	18
*kH* = 1	2383.603 2666.857 3020.240	2383.603 2666.858 3020.240	2383.603 2666.858 3020.240	2383.603 2666.859 3020.240	2383.603 2666.859 3020.240	2383.603 2666.859 3020.240
*kH* = 2	2379.092 2646.121 2964.807	2379.108 2646.171 2964.824	2379.109 2646.176 2964.835	2379.109 2646.176 2964.835	2379.109 2646.176 2964.835	2379.109 2646.176 2964.835
*kH* = 4	2375.942 2623.528 2873.811 3136.946	2376.246 2624.465 2873.338 3129.538	2376.322 2624.881 2873.912 3129.320	2376.338 2624.978 2874.013 3129.142	2376.341 2624.994 2874.031 3129.117	2376.341 2624.994 2874.030 3129.117

**Table 5:** Values of *P*(*i*,*j*).

	*j*	5	6	7	8
*i*					
3		−32,090,076,944.221519	10,660,198,006.060303	14.9250551593	19.5971240622
4		0.9727010301	0.9908836741	0	0
5		14.9250551593	19.5971240622	−0.0000000076	−0.00000001
6		−1,121,134,516.9505436	−362,357,971.9056837	1.5430806348	1.1752011936

**Table 6:** Values of *Q*(*i*,*j*) with the truncation *n* = 20.

	*j*	5	6	7	8
*i*					
3		−3,209,0076,944.221516	10,660,198,006.060305	14.9250551593	19.5971240622
4		0.9727010301	0.9908836741	0.0	0.0
5		14.9250551593	19.5971240622	−0.0000000076	−0.00000001
6		−1,121,334,516.9505434	−362,357,971.90568332	1.5430806348	1.1752011936

## Data Availability

The original contributions presented in this study are included in the article. Further inquiries can be directed to the corresponding author.

## References

[1] Jakoby B., Vellekoop M.J. (1997). Properties of Love waves: Applications in sensors. Smart Mater. Struct..

[2] Choudhari A., Rube M., Sadli I., Sebeloue M., Tamarin O., Dejous C. (2024). Love-wave acoustic sensors behavior in complex liquids: Multiparameter sensing using acoustic and electrical signals. IEEE Sens. J..

[3] Ghosh M., Dey S., Mitra S. (2025). Love-wave group-velocity tomography of India-Tibet: Insights into radially anisotropic S-wave velocity structure. Geophys. J. Int..

[4] Kshitish C.M., Alneamy A., Das A., Guha S., Tharwan M. (2026). Dispersion characteristics of Love-type waves in PFRC-piezoelectric interfaces with electric membrane and classical spring coupling. Int. J. Mech. Mater. Des..

[5] Majhi D.K., Kumar M., Rajak B.P., Vishwakarma S.K. (2025). Effect of initial stress and inhomogeneity on the Love wave propagation in an inhomogeneous composite structure. Acta Mech..

[6] Pranav P., Rahul A.K. (2025). Love-type wave propagation in a heterogeneous medium between a liquid and a fractured porous half-space: A WKB and variable separation approach. Eur. J. Mech. A Solids.

[7] Assouar M.B., Kirsch P., Alnot P. (2009). New Love wave liquid sensor operating at 2 GHz using an integrated micro-flow channel. Meas. Sci. Technol..

[8] Piliposian G.T., Danoyan Z.N. (2009). Surface electro-elastic Love waves in a layered structure with a piezoelectric substrate and two isotropic layers. Int. J. Solids Struct..

[9] Chen X., Liu D. (2009). Temperature stability of ZnO-based Love wave biosensor with SiO_2_ buffer layer. Sens. Actuators A Phys..

[10] Mirhosseini M.M., Khordad R. (2025). Propagation of Love waves in a three-layer structure: Substrate-polyurethane-piezoelectric. Int. J. Mod. Phys. C.

[11] Chen X., Liu D., Chen J., Wang G. (2009). The effect of a SiO_2_ layer on the performance of a ZnO-based SAW device for high sensitivity biosensor applications. Smart Mater. Struct..

[12] Cao X.S., Jin F., Jeon I. (2009). Rayleigh surface wave in a piezoelectric wafer with subsurface damage. Appl. Phys. Lett..

[13] Cho H., Hara Y., Matsuo T. (2014). Evaluation of the thickness and bond quality of three-layered media using zero-group-velocity Lamb waves. J. Phys. Conf. Ser..

[14] Li P., Shan S., Wen F., Cheng L. (2019). A fully-coupled dynamic model for the fundamental shear horizontal wave generation in a PZT activated SHM system. Mech. Syst. Signal Process..

[15] Jang H.L., Lee S.H., Kang L.H. (2025). Non-destructive testing for thickness measurement of the adhesive layer and defect detection in ceramic-metal bonded structures using THz waves. Struct. Health Monit. Int. J..

[16] Wu T.-T., Chen Y.-C. (1996). Dispersion of laser generated surface waves in an epoxy-bonded layered medium. Ultrasonics.

[17] Wu T.-T., Liu Y.-H. (1999). Inverse determinations of thickness and elastic properties of a bonding layer using laser-generated surface waves. Ultrasonics.

[18] Qu Y., Jin F., Yang J. (2020). Effects of mechanical fields on mobile charges in a composite beam of flexoelectric dielectrics and semiconductors. J. Appl. Phys..

[19] Li P., Jin F., Lu T.J. (2012). A three-layer structure model for the effect of a soft middle layer on Love waves propagating in layered piezoelectric systems. Acta Mech. Sin..

[20] Kumari A., Kundu S. (2025). Dispersion characteristics of Rayleigh waves in a corrugated nonlocal fiber-reinforced layer bonded to a nonlocal substrate with imperfect interface. Math. Methods Appl. Sci..

[21] Achenbach J.D., Balogun O. (2010). Anti-plane surface waves on a half-space with depth-dependent properties. Wave Motion.

[22] Sahu S.A., Nirwal S. (2021). An asymptotic approximation of Love wave frequency in a piezo-composite structure: WKB approach. Waves Random Complex Media.

[23] Issam B.S., Yassine W., Ghozlen M.H.B. (2011). Love waves in functionally graded piezoelectric materials by stiffness matrix method. Ultrasonics.

[24] Yan B.W., Gao Q. (2025). An accurate and efficient method based on the dynamic stiffness matrix for analyzing wave propagation in defective lattice structures. Int. J. Solids Struct..

[25] Talebitooti R., Ahmadi R., Shojaeefard M.H. (2015). Three-dimensional wave propagation on orthotropic cylindrical shells with arbitrary thickness considering state space method. Compos. Struct..

[26] Qu Y., Pan E., Zhu F., Jin F., Roy A.K. (2023). Modeling thermoelectric effects in piezoelectric semiconductors: New fully coupled mechanisms for mechanically manipulated heat flux and refrigeration. Int. J. Eng. Sci..

[27] Zhu J., Ye G.R., Xiang Y.Q., Chen W.Q. (2011). Recursive formulae for wave propagation analysis of FGM elastic plates via reverberation-ray matrix method. Compos. Struct..

[28] Tang D., Pang F., Zhang Z., Li L. (2021). Flexural wave propagation and attenuation through Timoshenko beam coupled with periodic resonators by the method of reverberation-ray matrix. Eur. J. Mech. A Solids.

[29] Othmani C., Njeh A., Ghozlen M.H.B. (2018). Influences of anisotropic fiber-reinforced composite media properties on fundamental guided wave mode behavior: A Legendre polynomial approach. Aerosp. Sci. Technol..

[30] Yu J., Wang X., Zhang X., Li Z., Li F. (2022). An analytical integration Legendre polynomial series approach for Lamb waves in fractional order thermoelastic multilayered plates. Math. Methods Appl. Sci..

[31] Zhang X., Dubois F., Sauvat N., Takarli M. (2022). Hybrid numerical method for the ultrasonic wave propagation velocity in orthotropic materials. Wood Sci. Technol..

[32] Pan W., Sun X., Wu L., Yang K., Tang N. (2019). Damage detection of asphalt concrete using piezo-ultrasonic wave technology. Materials.

[33] Zhao Q., Li F., Liu J. (2024). Analytical modeling of wave absorption performance in gradient graphene/polymer nanocomposites. Materials.

[34] Xu Z., Wang T., Li P., Wang B., Qian Z., Maruyama T., Zhu F., Kuznetsova I. (2025). Analysis of guided wave propagation in highly damped viscoelastic multilayered composite structures using the Biot model. Ultrasonics.

[35] Qu Y., Pan E., Zhu F., Deng Q. (2025). A thermodynamically consistent theory for flexoelectronics: Interaction between strain gradient and electric current in flexoelectric semiconductors. Int. J. Eng. Sci..

[36] Miao H., Li F. (2021). Shear horizontal wave transducers for structural health monitoring and nondestructive testing: A review. Ultrasonics.

[37] Li D., Li S., Zhang C., Chen W. (2023). Propagation characteristics of shear horizontal waves in piezoelectric semiconductor nanoplates incorporating surface effect. Int. J. Mech. Sci..

[38] Qian Z., Jin F., Lu T., Kishimoto K. (2008). Transverse surface waves in functionally graded piezoelectric materials with exponential variation. Smart Mater. Struct..

[39] Li P., Jin F., Qian Z. (2013). Propagation of the Bleustein-Gulyaev waves in a functionally graded transversely isotropic electro-magneto-elastic half-space. Eur. J. Mech. A/Solids.

[40] Yamada K., Sakamura J., Nakamura K. (2001). Equivalent network representation for thickness vibration modes in piezoelectric plates with a linearly graded parameter. IEEE Trans. Ultrason. Ferroelectr. Freq. Control.

[41] Cao X., Jin F., Jeon I., Lu T.J. (2009). Propagation of Love waves in a functionally graded piezoelectric material (FGPM) layered composite system. Int. J. Solids Struct..

[42] Qu Z., Cao X., Shen X. (2018). Properties of Love waves in functional graded saturated material. Materials.

[43] Li P., Biwa S. (2020). Flexural waves in a periodic non-uniform Euler-Bernoulli beam: Analysis for arbitrary contour profiles and applications to wave control. Int. J. Mech. Sci..

[44] Li P., Lu M., Qian Z., Kuznetsova I., Kolesov V., Ma T. (2022). Flexural wave control via the profile modulation of non-uniform Timoshenko beams. Mech. Mater..

[45] Jiang S., Jiang Q., Li X.F., Guo S.H., Zhou H.G., Yang J.S. (2006). Piezoelectromagnetic waves in a ceramic plate between two ceramic half-spaces. Int. J. Solids Struct..

[46] Liu J.X., Fang D.N., Wei W.Y., Zhao X.F. (2008). Love waves in layered piezoelectric/piezomagnetic structures. J. Sound Vib..

[47] Liu J., Cao X.S., Wang Z.K. (2007). Propagation of Love waves in a smart functionally graded piezoelectric composite structure. Smart Mater. Struct..

[48] Li X.Y., Wang Z.K., Huang S.H. (2004). Love waves in functionally graded piezoelectric materials. Int. J. Solids Struct..

